# Outlines of Physiology, with an Appendix, Containing Heads of Lectures on Pathology and Therapeutics

**Published:** 1831

**Authors:** 


OUTLINES OF PHYSIOLOGY, WITH AN APPENDIX, CONTAINING HEADS OF LECTURES ON PATHOLOGY AND THERAPEUTICS. By *W. P. Alison*, M.D., Professor of the Institutes of Medicine in the University of Edinburgh. 8vo. pp. 452. Blackwood, Edinburgh, & Cadell, London, 1831.

IN taking up this work, we trust that neither the author nor the reader will anticipate, that we purpose bringing before the one the substance of an elementary treatise on physiology, or that it is our intention to scrutinize, with the minuteness of very detailed criticism, the claims of the other to the merits of an able and advantageous performance. We are sick and sorry when we say it, but physiology in any form is among the least vendible articles which we take to market in these miserly days of greedy money-making, and although we might be discharging a wholesome and honourable duty, were we to stop and reason with our readers upon their shameful indifference to physiological discoveries and physiological discoverers, yet if it be true, as we believe it is, that all our reasoning on this subject would be utterly lost upon nine-tenths of those whom we address, we must feel it to be more imperatively our duty, both as regards them and ourselves, not to enter into any dispute upon the matter. The day is fast coming, when a knowledge of physiology will be considered somewhat more substantial than an ornamental fringe in the texture of a physician's education, and when to be able to discover and to remedy deranged function will be considered as indispensable, as to detect and to renovate impaired structure; but at present we fear that we must suffer the *amor nummi*, which is so epidemically prevalent, to subside, ere we can raise our voice to any very audible elevation in behalf of one of the most useful, yet one of the least cultivated sciences in medicine. He, who is determined to learn nothing which cannot directly increase his revenue, will not be very likely to hearken to our admonitions, and he, who can study physiology on its own account, does not require them; so that in passing on without further preface to the work before us, we shall not defraud the former by declining to advise where exhortation would not be received, and we may please the latter by not attempting to tender advice where it is felt to be unnecessary.

These Outlines of Physiology contain eighteen sections, to which is added an appendix comprehending lectures on pathology and therapeutics, which, it appears, are to constitute the ground-work of another volume. The first eleven sections are devoted to the grand nutritive functions of organic life; and in the remaining seven, which engross nearly half the book, are considered the more purely animal and metaphysical faculties. In a few important preliminary observations, with which he prefaces his description of the "laws of vital contractions," the Dr. observes—

"As the phenomena of life are seen only in bodies more or less organized, it has been conjectured that they depend merely on organization; but when we inquire how organization has been effected, we find that it implies in every instance, where we can observe it, the previous existence of vitality; and therefore must be regarded as one of its effects, not as its cause.

On the other hand, the supposition entertained by others, of a *material substance*, such as an etherial or subtile fluid, superadded to organization during life, and producing the phenomena of life, is both unsupported by evidence, and useless in the explanation of facts.

Setting aside both these hypotheses, we hold that all physiological inquiries are intended only to *ascertain the conditions, under which the various phenomena of Life take place*, and naturally terminate in a reference to certain *Laws of Vitality*, or ultimate facts in this department of nature; just as the investigation and explanation of phenomena in the inanimate world terminate in a reference to certain Laws of Motion, of Gravitation, of Chemical Affinity, &c. Of such first principles in science we can give no other account, than that they depend on the will of the AUTHOR OF NATURE; but the determination of such first principles is the main object, and the applications of them constitute the details, of all sciences; and every science is thus mainly conversant with principles *peculiar to itself*." 4.

This distinction between vitality, as the cause of organization and not its effect, has been sadly overlooked, and the oversight has led many physiologists into errors of the most serious description. It is true that we can know little if any thing of life, and therefore it may be vain to study it. Its phenomena are palpable, but its essence is veiled in mystery. Its operations are intelligible, but its seat and essence are utterly unknown. All this, no doubt, is true, and it is to be feared that the very admission of these facts has encouraged some into the suspicion, that life is a mere congregation of phenomena—the sum of certain vital processes—a mere name—a concise and convenient term whereby to express an assemblage of ascertained and acknowledged circumstances. Such physiologists deny its entity. They call it nothing. They hold that in the strict language of philosophy it is a phantom, a fancy, a creature of convenience, and that we have no reason for thinking otherwise, until forsooth our dissection-knife have revealed to us its texture, and until we can measure and weigh and handle it as we do any other piece of material substance.

Now, it may be philosophy, which cautions us not to dare to be wise beyond what is certainly known; but it is incredulity, which prevents us from believing all that is clearly demonstrated; and we have just as much evidence of the entity of life, as we have of soul, or of spirit, or of any other agent, whose existence is known only through the medium of its operations. It is life, which deposits, organizes and supports the constituent textures of the animal frame; it is not the constituent textures of the animal frame which generate life. Brain and nerve, blood-vessel and muscle are secreted under the agency of life, are organized by the power of life, and are renovated when impaired by the pure instrumentality of life. No animal process can commence in the fœtus, or continue in the adult without the operation of this active principle; and it is a sad confusion of all etiological relations, which ascribes to the passive and naked effect the very existence of the agent, to which this effect owes its being. Life is antecedent to organization, and organization when properly understood implies its pre-existence. Life generates life, and although organization is found in inseparable connexion with life in the present state of being, we see no necessary brotherhood between them, for it is not only conceivable but probable, that life exists without organization, although organization cannot exist without life. While, therefore, it may be philosophy to predicate nothing of life as an independent entity, because, never having seen it separate from matter, we know nothing of it in the abstract; it is neither philosophical nor courageous to permit this fear of knowing too much to keep us ignorant of every thing, and because we are unable to analyze the vital principle with the precision of a chemical result, to dismiss the subject from consideration by denying it existence altogether.

"The explanation of many of the phenomena of living animals is still very imperfect: but enough has been done to shew, that the principal laws regulating these phenomena must be ranked under three heads: 1. Those of *Vital Contractions*, by which the visible movements of living animals are chiefly effected: 2. Those of *Vital Affinities*, by which the chemical changes peculiar to living animals are determined, and their physical structure maintained: 3. Those of *Nervous Actions*, by which the physical changes in living animals are placed in connexion with Mental phenomena, and subjected to the control of Mental acts.

Of these, the vital affinities are perhaps the most general and the most fundamental; but they are the least understood, and, in the higher animals at least, their exercise is dependent on internal and vital contractions; and the laws of these contractions are, therefore, properly to be considered first." 5.

Our author's views upon the connexion, which is supposed to exist between muscular action and nervous energy, although not solitary, are somewhat peculiar. It is well known that, according to some, muscular fibre depends for its faculty of contraction upon some influence derived from the brain and spinal cord; while it is maintained by others that, although muscle does not derive this power from nervous energy, yet it is so far dependent on it for the existence and exercise of this faculty, that all stimuli, which excite muscular fibre to contraction, act upon it through the nervous filaments with which it is supplied. The Professor strongly opposes both these views, and the arguments upon which he rests his opposition, it must be admitted, are neither few nor feeble. In contradiction to the first doctrine he contends, that contractility is found in vegetable and in the lowest tribes of animal life, where no traces of a nervous system are discoverable;—that children have been born without brain or spinal cord, and yet their muscular system was in undiminished possession of this contractile power;—that circulation can be continued in warm-blooded animals after both the brain and spinal cord are destroyed, by keeping up an artificial action within the lungs;—that after the nerves, which supply voluntary muscles, are divided, although the operation relieves such muscles from their subjection to the will, yet they may afterwards be made to contract by applying stimuli to themselves, and contractions may be excited as long in such muscles as in those, whose communication with the brain and cord is entire; and, lastly, that neither the heart, nor any other strictly involuntary muscle, can be deprived of its contractility by cutting the nerves which immediately supply them. Such are the Doctor's leading objections against the first view. His objections to the second we shall give in his own terms;—

"To the *second* of the theories above stated, it appears a sufficient objection to state, that our only reason for supposing an intervention of nerves to be concerned in muscular contraction, is the excitation of that contraction by stimuli applied to nerves. But a conclusion which is rested on this fact, must be limited to the cases in which this fact holds good. Now, there are *many muscles* (viz. all or almost all those that are destined to *involuntary motion* only), which, although exceedingly irritable, *are not exciteable by mechanical irritation of their nerves.* Even Galvanism, applied exclusively to the nerves of these muscles, has generally failed to excite them; and in the instances where galvanism, so applied, has had some effect, it appears probable that the nerves acted only as conductors of the galvanism to the muscular fibres themselves. When experience shews, that some muscles are excitable by irritation of their nerves, and others not, we cannot acquiesce in the proposition that nerves furnish a condition essential to the irritation and vital action of muscles in general.

We must therefore set aside both the hypotheses above mentioned; and in so doing, we necessarily limit the meaning of the terms 'Nervous Energy, Nervous Influence, Innervation,' &c. in reference to the connexion of vital movements with nerves, to a degree, of which many of those who use these terms do not seem to be aware.

It remains that, on this point, we acquiesce in the judgment of Haller*, as the only generalization yet admissible, of the facts known in regard to it, viz. That the *vital power of Muscles is inherent in themselves,* and in no case dependent on Nerves; but is liable to affection in two distinct ways, by changes in certain parts of the nervous system, whether these are produced by physical or mental causes;—being *directly excited in many muscles,* and *increased or diminished,* or *variously altered,* probably *in all muscles,* by such changes." 15.

We have always considered the experiments of Wilson Philip, and of those physiologists who have endeavoured to establish the independence of the blood-vessel upon the nervous system, as obnoxious to one common objection, which has been seldom if at all sufficiently urged. When they have separated the heart from its nerves, and removed it from the body, they have imagined that, because its auricles and ventricles have continued to act for some time, the muscular tissue of this organ is endowed with a

* "See particularly Elem. Physiol. lib. 17, sec. 2, § 7."

*vis insita*, peculiar to itself, to which it is indebted for its power of contraction. But, without attempting to give either a name or a nature to the nervous energy; without calling it a fluid, or prescribing any specific limits beyond which it may cease to operate, is it not natural to imagine, that if it exercise any influence whatever upon the circulating system, that influence must be more fixed and permanent than to be instantaneously annihilated by any experiment, which does not annihilate the nervous system itself? When the heart of a living animal is removed from the body, the operation does not separate this organ from the supply of nervous influence which it is supposed to have contained before the separation was effected;—it only cuts it off from the source and continuance of this supply. The nervous influence, which it *has* received and which it *does* contain, is not annihilated by the experiment, and therefore until this supply shall have been exhausted by the activity of the organ, and until it can be shewn that this activity continues to exist *after* the period of this exhaustion has been completed, it should not be said that the heart can act without nerves, or is independent of the nervous system. After the heart is removed from the body the capillary arteries continue to propel their contents into the veins, the lacteal and lymphatics carry onwards to the heart their nutrient current, muscular fibre contracts and oscillates for a considerable period, and every part of the frame, which is endowed with a moderate share of sensibility, not only answers for a time to the application of stimuli, but continues to exhibit some of the properties of life, or, as the Doctor would say, "vital contractility" after the general system has expired. All this proves—not that the capillary arteries and absorbents and muscles can discharge their respective functions without nerves—but that the nervous influence—whatever it may be—with which these textures are supplied, operates within them for some time after apparent death, and endowes them as it were with a *post-vitam* existence of their own. What seems to us to go far in confirmation of this view is the fact, that this excitability survives the general extinction of life for a very short period in any case; that after death by lightning this excitability is simultaneously and equally destroyed with every other animal function; and that it may be almost instantly exhausted by applying to the excited texture electricity, galvanism, or such other active stimuli, as are known strongly to excite and rapidly to exhaust nervous energy.

That muscular texture possesses a vis insita peculiar to itself, is, we think, extremely problematical. It is no argument to the contrary to maintain that vegetables are endowed with contractility, although we cannot discover in their composition nervous tissue; nor that the lower tribes of animals exhibit mature and contractile power, without any ascertained supply of nerves. The lacteals existed in man from the days of Adam till the time of Acellius, yet they remained undiscovered; and until Rudbeck demonstrated the lymphatics at a still later period, that extensive and important system of vessels was utterly unknown. Shall we say, then, that in any animal system, exhibiting muscular fibre, blood-vessel, contractility and motion, there are no nerves, merely because we cannot see them; or that in the mimosa sensitiva, which betrays as much irritability of temperament as the most delicate and nervous constitution, its sensibility and contractile power are wholly independent of every thing like nervous influence? Any argument drawn from this source is purely negative, being no more than an *argumentum ad igno-*

*rantiam*, and when we consider the extreme darkness in which we are upon points of the greatest interest in physiology, we can build no very enduring opposition to one theory on the basis of another, which is supported not by what we know, but by what we do not know.

The two original objections, which were advanced against the doctrine which we are now considering in the days of Haller—that the heart sympathizes with emotions of the mind, and is abundantly supplied with nerves issuing from more sources than one—possess the same cogency at the present hour which they had at first; and, although the amount of our information upon this point may not entitle us to specify the precise limits of connexion, which obtain between the nervous and blood-vessel systems, still that there is a connexion and one of very considerable intimacy, many facts and very many phenomena conspire to prove. The great difficulty in this question—with us at least—is clearly to ascertain that department of the nervous system, which more immediately influences the heart and arteries, and the exact extent to which this influence operates. There is reason to believe that the heart exists in the fœtus prior to the brain, and that the majority of the arteries are deposited before the spinal marrow can be discovered. If this be so, the heart and arteries should be more immediately connected with the ganglionic system, and all our far-famed experiments upon the brain and spinal cord—our slicing and pithing and pricking and singeing these organs—to ascertain what effects such treatment might produce upon the action of the heart, must go for nothing, being directed towards the wrong quarter. The heart we know is one of the principal muscles of the organic life, and *a priori* it were reasonable to imagine that its nerves would be principally derived from that system which mainly supplies the organs of interior life; and if the pre-existence of the heart and arteries can be fully established, the circulation must be greatly independent of this system, and so far the school of physiologists, to which Dr. Alison pertains, may be borne out in their opinions. The experiments of Le Gallois, no doubt, prove sufficiently clearly that the heart can act for a considerable time after the brain has been destroyed; and Wilson Philip's experiments also prove, that both the brain and spinal marrow may be broken down and scorched with a hot wire, and yet the heart's action continue, provided artificial respiration be established; but the ganglionic system still remains to be considered, and we suspect that the important fact will soon be established, that the heart's life, motion and energy depend immediately, if not exclusively, on nerves belonging to the ganglionic or organic system. Brachet, in his late admirable work upon the functions of the ganglionic system, has in our opinion done much to settle this point; but as a more special and a fitter opportunity will, probably, soon offer to the writer of these remarks for entering with more minuteness into this highly interesting subject, we shall decline to add to the few general observations, which have been thrown out, *en passant*, in noticing this portion of the Professor's Outlines.

It is now, we believe, very generally admitted, that absorption is not exclusively performed by lacteals and lymphatics; but that this function is at least occasionally exercised by capillary veins. Dr. Alison strongly advocates this extra faculty of the venous capillaries.

"The experiments of HUNTER, made by exposing and isolating small portions of the intestines of living animals, filling them with different fluids, chiefly milk and a solution of indigo, and then examining the contents of the lacteals, and of the veins leading from these, may be allowed to prove two points: *1st*, That absorption, at least of milk, and probably of other fluids, different from chyle, took place in his trials by the lacteals; *2dly*, That no absorption could be ascertained, in his trials, to have taken place by the veins.

The first of these, which is a positive observation, although opposed to the results obtained by MAGENDIE and others, agrees with the results of many other experiments, by LISTER, HALLER, BLUMENBACH, TIEDEMANN and GMELIN, LAWRENCE and COATES, and FODERA, in which it appeared that a certain portion of different fluids, introduced into the intestines, was taken up by the lacteals; and the possibility of their absorbing fluids different from chyle may, therefore, be held to be decided. But the second observation of Mr. Hunter, which is a negative one, is quite an insufficient ground for the general conclusion, that veins do not absorb; and the reality of venous absorption is now put beyond all doubt, by the positive observation of many physiologists, particularly by the following.

1. The experiments of Sir E. HOME and Mr. BRODIE* prove, that when the great lymphatic trunks are tied in warm-blooded animals, substances injected into the stomach quickly find their way into the circulation, and may be detected in the urine.

2. Experiments made by MAGENDIE, FLANDRIN, TIEDEMANN and GMELIN, and others, prove that odoriferous substances, known by their smell, and saline substances, indicated by their tests, after being taken into the stomach, are detected in the veins on the mesentery, both larger and smaller, and in the vena portæ, much more than in the lacteals and thoracic duct.

3. Experiments made by MAGENDIE, prove that a poison introduced into an isolated portion of intestine, communicating with the rest of the body only by an artery and vein, or into the cellular texture of a similarly isolated limb, acts in the usual way, and nearly in the usual time, when the circulation is free.

4. In experiments made by SEGALAS, it appeared that a poison introduced into a portion of intestine between two ligatures, failed of effect as long as the artery and vein leading to that portion were tied, although the lacteals and other textures were uninjured, but took effect as soon as the circulation was set free.†

5. In experiments made by Professor MAYER, it appeared that saline substances introduced in small quantity into the bronchiæ of animals, found their way very quickly into the blood, although the thoracic duct was tied, and were detected in the left side of the heart much sooner than in the right side.‡

6. In experiments by FODERA,§ it appeared that two saline solutions, applied to the inner and outer membrane of an isolated portion of intestine in a living animal, *were united in the small veins* leading directly from that portion of intestine.

7. In experiments by MAGENDIE, it appeared that a poison applied to an isolated vein, with all precautions to avoid contact with other textures, or even to an isolated artery, gradually transuded into the interior of the vessel, and then produced its usual effects.

8. In cases of disease where large deposits of morbid matter have taken place within a short time,—in cases of Suppuration, of Fungus Hæmatodes, and of Melanosis, the veins of the affected parts have been found loaded with the morbid matter, more generally than the absorbents.

It would appear, therefore, that the veins are concerned in the function of

---

* Phil. Trans. 1808. † Journal de Physiologie, 1822.

‡ Bibliotheque Universelle, Jan. 1818.

§ Recherches Experimentales sur l'Absorption et l'Exhalation.

absorption in all the following ways:—1. They themselves absorb, chiefly by their smallest branches, at least fluid matters. 2. The contents of the lacteals and lymphatics are probably partially intermixed with those of the veins in lymphatic glands. 3. Some of the smaller lymphatic trunks terminate in veins. 4. The largest lymphatic trunks terminate in the great veins of the neck." 84.

Since the appearance of Wilson Philip's work on the vital functions, it has been very fashionable to believe, that nervous influence is identical with the galvanic fluid, or that the relationship between the laws and properties of these two principles is so close, that the effects which they produce upon the animal frame are strikingly alike. Although there may be more than plausible appearances to countenance this theory, it appears to us very far from being so settled by demonstrative experiments, as to warrant the general adoption which it has received from the profession. Galvanic excitement is the strongest stimulant which can be applied to the muscular fibre, and after all other forms of stimuli have ceased to operate upon the dead body, an electric shock can still awaken its irritability, and stimulate the muscles into violent contortions. But if galvanic be the same with nervous influence, or even a tolerable substitute, why can we not preserve the irritability of muscle after it has been separated from its nerves, by means of this agent for an unlimited period? Why does the stomach digest food for a very short period only, after section of the eighth pair of nerves, although it be regularly supplied with galvanic influence; and why will muscular irritability ultimately cease, although it be unremittingly excited by electricity? As long as galvanism is applied, contractility and secretion should proceed if these functions depend upon the action of nerves, and if galvanic influence be the same with nervous energy; but in despite of this stimulus, they speedily cease, and not only cease, but, what is strikingly worthy of attention, cease more speedily in cases where this stimulus has been applied, than in those where no artificial efforts have been made to prevent its exhaustion. Although we are very far from believing in many of Dr. Calvert Holland's conclusions, in his Experimental Inquiry into the Laws of Organic and Animal Life, still the experiments, which he has performed upon the par vagum, in our opinion wholly overturn the conclusions of Wilson Philip in favor of the identity of galvanic and nervous influence, which he has drawn from experiments upon the same nerve. It is now quite certain that the stomach can digest its contents after section of this nerve, or even excision of a part of it, if the trachea be previously divided, or—what we have found equally sufficient—if an aperture be made in the windpipe, large enough to contain a tube of the size of an ordinary female catheter. It would appear, therefore, that indigestion follows section of the eighth pair, without this precaution, principally, if not wholly from the derangement which occurs in the pulmonary functions; and that galvanism, in restoring the digestive process, acts only as a local stimulant which may be entirely dispensed with, if the lungs be suffered to carry on their functions without impediment.

Again, if galvanism and nervous influence have any properties in common, how does it happen that the former can act upon muscular fibre through some nerves only, and not through all? From the experiments of Haller, Fontana, Bichat and Mayo, it would appear that the heart and intestines are unaffected by irritating the nerves which immediately supply them; and Bell, Magendie and Beclard have shown that scarcely any muscular contractions can be excited by the posterior portions of the spinal nerves or the ganglionic department of the fifth nerve, although strong muscular action may in this way be elicited through the anterior spinal nerves, and through the third, fourth, sixth, eighth, ninth, and part of the fifth and seventh central nerves. Were galvanic fluid generated in the human body by the contact of nervous with muscular texture, it should be elicited from all nerves, if not equally yet in some degree; and could galvanic fluid supply the place of nervous energy, it should not happen that one class of nerves are obedient to such stimulation, while another class, which are equally indispensable to muscular action, wholly refuse to convey to the textures they supply the galvanic stimulus. The Doctor does not devote much space to this subject, but we are glad to find that the observations, which he makes upon it, are characterized by that prudent discrimination and dislike of theory, which mark not only the present volume, but all the preceding investigations of the author.

"Although we gave reasons for thinking that Secretion and Nutrition are truly independent of nerves, yet several facts show, that physical impressions on, or injuries of, the nervous system, materially and variously *influence these functions*, as well as the circulation in the small vessels, which are their seat. This kind of influence of physical impressions on nervous matter, is also imperfectly understood, and not easily distinguished from the effects of mental acts; but it seems exemplified in the following instances:

1. The effect of section of the eighth nerve in the neck, in not merely suspending the secretion of gastric juice at the stomach (which is a somewhat ambiguous case), but also in exciting a degree of inflammatory action there; in preventing the usual effusion of mucus in the intestines, even when arsenic has been swallowed;* and, on the other hand, in exciting inflammation, and increasing the mucous secretion, in the lungs and bronchiæ.†

2. The effects (viz. inflammation, ulceration, and sloughing), produced on the eye-ball, and in some instances on the membrane of the nose, and on the gums, as was first ascertained by MAGENDIE, by section of the fifth nerve, which supplies these parts; and likewise, in a less degree, by section of the sympathetic nerve in the neck,‡—effects which have also been seen, in some cases in the human body, from disease of the fifth nerve.

3. The inflammatory condition, with increased and altered secretion, of the mucous membrane of the bladder, in many cases of paraplegia, dependent on injury of the spinal cord.

The diminished nutrition and diminished secretions (*e. g.* by the skin), often observed in a limb which has been for some time palsied, by section of its nerve, or disease of the brain, may be thought to illustrate the same point; but these effects are perhaps sufficiently explained by the total inactivity of such a limb.

It is to be observed, that such effects as those now stated, on secretion and nutrition, have been observed only in certain parts of the body, and chiefly from injury of certain of the nerves supplying these. These are the sentient nerves of the parts in question; and the secretions which are changed, are generally such as are habitually excited by irritations producing sensation. It is still

---

* Brodie, Phil. Trans. 1814.

† WILSON PHILIP, l. c. SWAN, Essay on the Connexion between the Action of the Heart and Arteries and the Nervous System.

‡ See DUPUY, Journal de Medecine, t. xxxvii.

doubtful, whether the whole of the effects of these injuries on secretion and nutrition may not be explained by these considerations.

These statements of physical phenomena, however, illustrate the very peculiar powers, known only by their effects on other parts of the animal frame, which the Nervous System in living animals possesses. Only one theory, in explanation of these powers, appears to deserve attention, viz. that which ascribes them to Galvanism, evolved in the animal frame, especially by the contact of nervous with muscular substance. It is known that, by the contact of these substances, galvanic phenomena, in a slight degree, may be produced; and that galvanism, however evolved, is a powerful stimulant of muscular contraction,—in an excessive degree, is a powerful sedative,—and has also appeared frequently to influence the capillary circulation and secretions.

It appeared also, in some experiments by Dr. Edwards, that when the nerve and muscle of a frog were laid on a good conductor of electricity, irritation of the nerve had much less effect in exciting the muscle, than when they were laid on a non-conductor; which he ascribed to the galvanism supposed to be excited in the nerve being carried off by the conductor of electricity in the former case, and therefore not affecting the muscle.*

But whatever be the true explanation of this fact, the following general objections may be stated to the Galvanic theory of Nervous actions, such as we have hitherto considered them.

1. The causes which excite, in the highest degree of intensity, those changes in nerves by which muscles are excited (*e. g.* such causes as bruising with a probe, or pricking with a pin), seem quite inadequate to the production of a sudden and powerful galvanic influence.

2. We have seen that these causes do not act on all nerves, and through them on all muscles which they supply, but only on the nerves of certain muscles, and only on certain of these nerves.

3. We have seen that the power of exciting muscular contraction is so far from residing in nervous substance in general, that it resides on one surface of the spinal cord, and not on the other,—nor in its centre; nay, it resides in one part of a nervous fibre, in the medulla oblongata, and not in another part of the same fibre, half an inch higher in the brain.

4. While the changes in the nervous system, which excite muscles to contraction, take place only in certain parts of the nervous system, those which exalt or depress the vital power of muscles, appear to take place especially in others, and therefore affect especially other muscles; and the same cause (*e. g.* a violent concussion) which produces one of these effects exclusively in one nerve, may produce the other in another nerve immediately adjoining it.

These facts seem sufficient to show, that if it be galvanism which enables nerves to act on muscles in the living body, it is galvanism excited by means, and subjected to laws, very different from what we observe in examining the galvanic phenomena of dead matter. And this is equivalent to saying, that nerves act on muscles in the living body, in virtue of certain *vital powers*." 150.

With this very short and imperfect notice of Dr. Alison's *Outlines* our readers must rest satisfied, as it is utterly impracticable to lay before them, in the present place, any adequate epitome of a volume of physiology, which is literally crowded with general statements and abstract facts. Written more immediately for students, and professedly as heads of lectures, it is not to be expected that much illustration and detail would be introduced, nor that the professor would often stop, while sketching the leading outlines of his course, to enter into original investigation or lengthened argument. When the subject is especially interesting he occasionally dilates, or when

* "Ann. des Sciences Naturelles, t. v."

the view in which he is indulging is considered open to dispute, plausible objections are anticipated and the leading arguments, by which it stands supported, are carefully laid down ; but even then conciseness and generalization are observed, and the reader is never permitted to forget that he is merely perusing the sketch of a performance, which the author intends to fill up with more minute research in another place. This much we have deemed it necessary to specify, as some superficial readers, forgetting the original aim of the writer, might consider many of his subjects unfinished, and some of them scarcely more than entered on. While, however, this work is to be regarded as an unfinished outline, which a course of lectures are intended to clothe with illustration and detail, it must be equally useful to the full grown physiologist, as to the mere student; for while it prepares the latter, by its general enunciations and leading statements, for entering into the more minute and mysterious labyrinths of the science of function, the former can advantageously refer to and depend upon it, for an able, concise, and correct exposition of the principal facts of his favourite science, which his more matured attainments can seldom fail to amplify with appropriate illustrations. It is written with the true spirit of an experimental philosopher. Unsupported theories are seldom noticed and never dwelt on; ascertained facts are carefully and fully stated; naked speculation is constantly avoided, and, although the Doctor is by no means innocent of novelty in some of his doctrines and opinions, yet he candidly adduces the authorities which exist against him, and briefly but fairly enumerates the arguments and facts by which they are opposed.

There can scarcely be a doubt, but that outlines so ably sketched will be ultimately filled up by the pen which drew them; and knowing as we do the industry, impartiality, and sound judgment of Dr. Alison, physiology could not but reap a harvest from his labors, were they only limited to a philosophical *digest* of our present system; of all the sciences, which are included within the curriculum of a medical man's education, none is so obnoxious to false facts and delusive fancies as physiology; and it were a question of no trifling difficulty in its present state to decide, whether he who adds to its real discoveries, be a greater benefactor than he, who sweeps from its table of contents all the crude imaginations which crowd and cumber it.

---

